# Self-regulation of students’ psychological states

**DOI:** 10.1192/j.eurpsy.2021.1929

**Published:** 2021-08-13

**Authors:** M. Kartasheva, A. Klimanova, A. Prokhorov, A. Chernov, M. Yusupov

**Affiliations:** General Psychology, Kazan Federal University, Kazan, Russian Federation

**Keywords:** mental state, self-regulation, student, consciousness

## Abstract

**Introduction:**

A system model of self-regulation of students’ psychological states has been developed. As the main elements, the model includes the relationship between states and characteristics of consciousness, external factors and regulatory actions in a certain time range.

**Objectives:**

To study conscious and unconscious methods of regulation of states in the relevant sections of the educational activities of students.

**Methods:**

98 students took part in this research, used different techniques of self-regulation and psychological states diagnosis.

**Results:**

The regulation of psychological states occurs unconsciously. The success of the applied methods is relative and depends on both educational and personal factors. The regulators of states are various personal qualities. These are reflection, metacognitive abilities, intelligence, as well as the general ability to self-regulate. We discovered the influence of the meaningfulness of life on the psychological states. In the structure of students’ states with a high level of meaningfulness of life an indicator of the general ability to self-regulation plays a central role.Indicators of emotional intelligence and locus of control characterize states of students with a low level of meaningfulness of life.

**Conclusions:**

It has been found that the level of reflection of students plays a mediating role in the interaction of psychological states and adaptation processes. Emotional comfort, internal control, and self-acceptance have the greatest impact on states. The research confirmed the hypothesis of reflexive regulation of psychological states depending on various types of reflection during the performance of creative tasks. The research was carried out with the financial support of the RFBR; project No.19-013-00325.

**Disclosure:**

No significant relationships.

